# Mapping the landscape of global programmes to evaluate health interventions in pregnancy: the need for harmonised approaches, standards and tools

**DOI:** 10.1136/bmjgh-2018-001053

**Published:** 2018-10-15

**Authors:** Patrick L F Zuber, Allisyn C Moran, Doris Chou, Françoise Renaud, Christine Halleux, Juan Pablo Peña-Rosas, Kavitha Viswanathan, Eve Lackritz, Robert Jakob, Elizabeth Mason, Smaragda Lamprianou, Christine Guillard-Maure

**Affiliations:** 1 Safety and Vigilance, Department of Essential Medicines and Health Products, World Health Organization, Geneva, Switzerland; 2 Department of Epidemiology, Monitoring and Evaluation, Department of Child and Adolescent Health and Development, World Health Organization, Geneva, Switzerland; 3 Adolescents and at-Risk Populations, Department of Reproductive Health and Research, World Health Organization, Geneva, Switzerland; 4 Strategic Information and Planning, Department of HIV/AIDS, World Health Organization, Geneva, Switzerland; 5 Intervention and Implementation Research, Special Programme for Research and Training in Tropical Diseases, World Health Organization, Geneva, Switzerland; 6 Evidence and Programme Guidance, Department of Nutrition for Health and Development, World Health Organization, Geneva, Switzerland; 7 Global Platform for Measurement and Accountability, Department of Information, Evidence and Research, World Health Organization, Geneva, Switzerland; 8 High Threat Pathogens, Department of Infectious Hazard Management, World Health Organization, Geneva, Switzerland; 9 Data Standards and Informatics, Department of Information, Evidence and Research, World Health Organization, Geneva, Switzerland; 10 Global Public Health SARL, Geneva, Switzerland

**Keywords:** maternal health, public health, vaccines

## Abstract

Pregnant women and their babies are among the populations most vulnerable to untoward health outcomes. Yet current standards for evaluating health interventions cannot be met during pregnancy because of lack of adequate evidence. The situation is even more concerning in low-income and middle-income countries, where the need for effective interventions is the greatest. Meeting the Sustainable Development Goals for health will require strengthened attention to maternal and child health. In this paper we examine ongoing initiatives aimed at improving the assessment of maternal interventions. We review current methodologies to monitor outcomes of maternal interventions and identify where harmonisation is needed. Based on this analysis we identify settings where different minimal data sets should be considered taking into consideration the clinical realities. Stronger coordination mechanisms and a roadmap to support harmonised monitoring of maternal interventions across programmes and partners, working on improving pregnancy and early childhood health events, will greatly enhance ability to generate evidence-based policies.

**Summary box**

- Health interventions during pregnancy are not subject to the same rigorous clinical evaluations as compared with those in non-pregnant women.
- Multiple initiatives are under way to improve pregnancy outcomes, which are reviewed here.
- The current diversity of terminologies, definitions and methods of assessment, and the variability of the data elements collected, do not allow comparability and meta-analysis.
- Multiplication of initiatives and tools results in loss of information where joining forces could avoid duplication of efforts while increasing the amount of available data.
- Harmonised metrics and methodologies should be developed to allow measuring safety and beneficial endpoints of pregnancy interventions in a comparable fashion.

## The need to monitor pregnancy outcomes

Equity towards health outcomes is a recognised public health priority,^1^ yet in resource-poor settings pregnant women and their infants remain among the most vulnerable.^2^ Although various interventions are available for pregnant women, concerns related to interventional research during pregnancy tend to limit the conduct of carefully designed clinical trials.^3^ As a result, the value of those interventions cannot be assessed with the same criteria and certainty of evidence as compared with most medical procedures. There is an urgent need to correct this situation through the generation of adequate prelicensure and postlicensure evidence. Monitoring pregnancy outcomes is a public health priority, not only to track maternal morbidity and disability, but to track adverse birth outcomes such as stillbirths, early neonatal deaths, and premature and low birthweight infants. Studies have shown that women with perinatal loss are more likely to experience depressive symptoms, guilt and prolonged grieving.^4^ In addition, there is evidence that babies born early or too small have a greater risk of developmental difficulties and chronic disease in adulthood.^5^ In this paper we examine a number of ongoing reproductive, maternal, newborn and child health initiatives in order to assess how synergies can be established for those efforts to generate useful and valid evidence to the benefit of pregnant women and their babies.

Vaccination in pregnancy illustrates ongoing challenges. Currently the WHO recommends the use of three vaccines: tetanus, pertussis and influenza.^6^ All three were licensed for other indications and were only later considered for their specific benefits during pregnancy. Therefore safety data are only available from postlicensure observational studies.^7^ At its December 2017 meeting, the Global Advisory Committee on Vaccine Safety—WHO’s independent expert advisory body for vaccine safety issues—agreed that minimal data elements would need to be assessed for their availability in different settings and for different types of studies. The Committee also indicated that efforts to enhance access to quality data on pregnancy outcomes would benefit the broad community of stakeholders working to improve the health of mothers and their infants.^8^

Consideration to the safety of health interventions in pregnancy requires innovative solutions to provide enhanced safety data collection and analysis (vigilance). We present an overview of approaches for data collection currently supported by the WHO programmes and other stakeholders, focusing on the needs of populations living in low-income and middle-income countries. We conducted an examination of the diversity of public health activities, terminologies and surveillance systems currently available for pregnancy-associated events and propose an integrative approach on a multiple stakeholders platform to support efficient coordination of maternal and child health global efforts.

Reproductive, maternal and neonatal health programmes are measuring numerous processes, events and outcomes during pregnancy and early childhood in order to determine the magnitude and distribution of those that require priority action, evaluate the impact of these actions and measure progress on the implementation of solutions through policies and programmes. The information can also be useful in understanding the diverse causes and determinants of the problem.^9^ Thus the use of the data can serve various purposes, including assessing the trade-off of benefits and harms. Although pregnancy interventions are meant to be beneficial, their potential risks should be measured with similar methods and definitions. For example, as several strategies effectively prevent preterm birth,^10^ it is also important to evaluate for each individual intervention if they do not also carry a possible risk for preterm birth. Therefore, regardless if an outcome would be measured as a benefit or a risk related to pregnancy interventions, the same harmonised definitions should apply.

## WHO programmes that address maternal, neonatal and child health

A number of WHO programmes include health promotion and preventive interventions for pregnant women. These programmes have global objectives, frequently with focus on low-income and middle-income countries, and are implemented at the country level in collaboration with multiple partners. The *Every Newborn Action Plan* 11 jointly developed by WHO and Unicef with support from several partners is a roadmap of strategic actions aiming to end preventable newborn mortality and stillbirths and to contribute to reducing maternal mortality and morbidity. It outlines the importance of collecting data on maternal and newborn survival and well-being. The annual report presents progress on the implementation of key interventions in countries with the highest rates for these adverse outcomes.^12^ Similarly, the strategies towards *Ending Preventable Maternal Mortality* provide targets to reduce global maternal mortality by 2030.^13^ *Maternal Death Surveillance and Response* 14 is a technical guide that emphasises the importance of tracking maternal mortality, and *Making Every Baby Count* 15 provides similar guidance addressing perinatal and neonatal mortality, assessing, diagnosing and assigning the causes of death, using information to improve the quality of care. There is ongoing work on tracking maternal morbidity through the use of common definitions and standardised tools. WHO participates in the global movement *Every Woman Every Child* and produced the *Indicator Framework for the WHO-led Global Strategy for Women’s, Children’s and Adolescents’ Health 2016–* *2030*, an initiative which defines indicators for monitoring maternal, child and adolescent outcomes.^16^ In 2012, the World Health Assembly (WHA) approved a comprehensive implementation plan on maternal, infant and young children nutrition that identified six global targets related to priority nutrition outcomes to be achieved by 2025. In collaboration with Unicef, WHO developed the *Tracking Tool* to help countries set their national targets and monitor progress.^17^ In 2014, member states approved the *Global Nutrition Monitoring Framework*,^18^ which includes processes and outcomes relevant to pregnant women.

Other general resources include the *WHO Open Smart Register Platform*, an open source mobile health platform that allows electronic registration and tracking of an entire population’s health,^19^ a toolkit of standards and norms for birth defects surveillance^20^ (jointly developed with the US Centers for Disease Control and Prevention (CDC) and the International Clearinghouse for Birth Defects Surveillance and Research), which has been implemented in several regions and countries,^21^ and a WHO central registry for the surveillance of drug safety in pregnancy for low-income and middle income countries.^22^ There are also activities specific to individual WHO programmes with interventions tailored for women during pregnancy. Some of these include evolving treatments and prevention for HIV and syphilis, tuberculosis and malaria, prevention of pre-eclampsia and other complications in pregnancy, or routine antenatal care. Vaccine research aims at optimising the use of existing vaccines, evaluate new ones for specific pregnancy indications and harmonise methodologies.

This diverse set of collaborative efforts faces many data limitations, both quantitative and qualitative, incomplete reporting and potentially misclassification. Comparability and potential for meta-analysis are limited by multiple definitions and methods of assessment, with great variability of the data elements collected. At the country level, these initiatives and programmes are often implemented differently in each context. The use of harmonised tools and methods, similar to what is promoted by WHO for disease surveillance and pharmacovigilance of vaccines for example, would provide considerable gains in order to strengthen the evidence base on maternal, newborn and child health outcomes.

## Examples of other global initiatives

Many other initiatives are under way to improve documentation of pregnancy outcomes illustrated by two large networks. Because pregnancies are diagnosed with high variability in different contexts, monitoring which relies on confirmed pregnancies (such as sentinel site-based surveillance) often misses a large number of early spontaneous abortions.^23 24^ One approach to reduce this detection gap is to capture health information independently from the routine healthcare. Health demographic surveillance sites (HDSS) collect systematic data from thousands of households in low-income and middle-income countries. When repeated at regular time intervals, these sites have data on cohorts that can allow for analysis of important predictors related to pregnancy, birth and death in particular. The INDEPTH Network (Better Health Information for Better Health Policy)^25^ currently includes 49 independent HDSS in urban and rural areas of 18 low-income and middle-income countries from Africa, Asia and Oceania regularly monitoring more than three million people. Pregnancy surveillance is an important aspect of the monitoring, with several core indicators regularly collected (pregnancy, stillbirth, live birth, neonatal death, infant deaths, maternal deaths, women delivering at a health facility, maternal age and causes of death). INDEPTH provides a valuable platform to test public health approaches and improve pregnancy outcomes tracking. Among many other examples, the Core Outcomes in Women’s and Newborn Health initiative^26^ is a consortium of obstetricians that promotes reporting of results according to prespecified core outcomes. To date 13 sets of core outcomes have been published providing trial designers with outcome sets as a basis for their protocols.

## Terminologies

**Figure 1:**
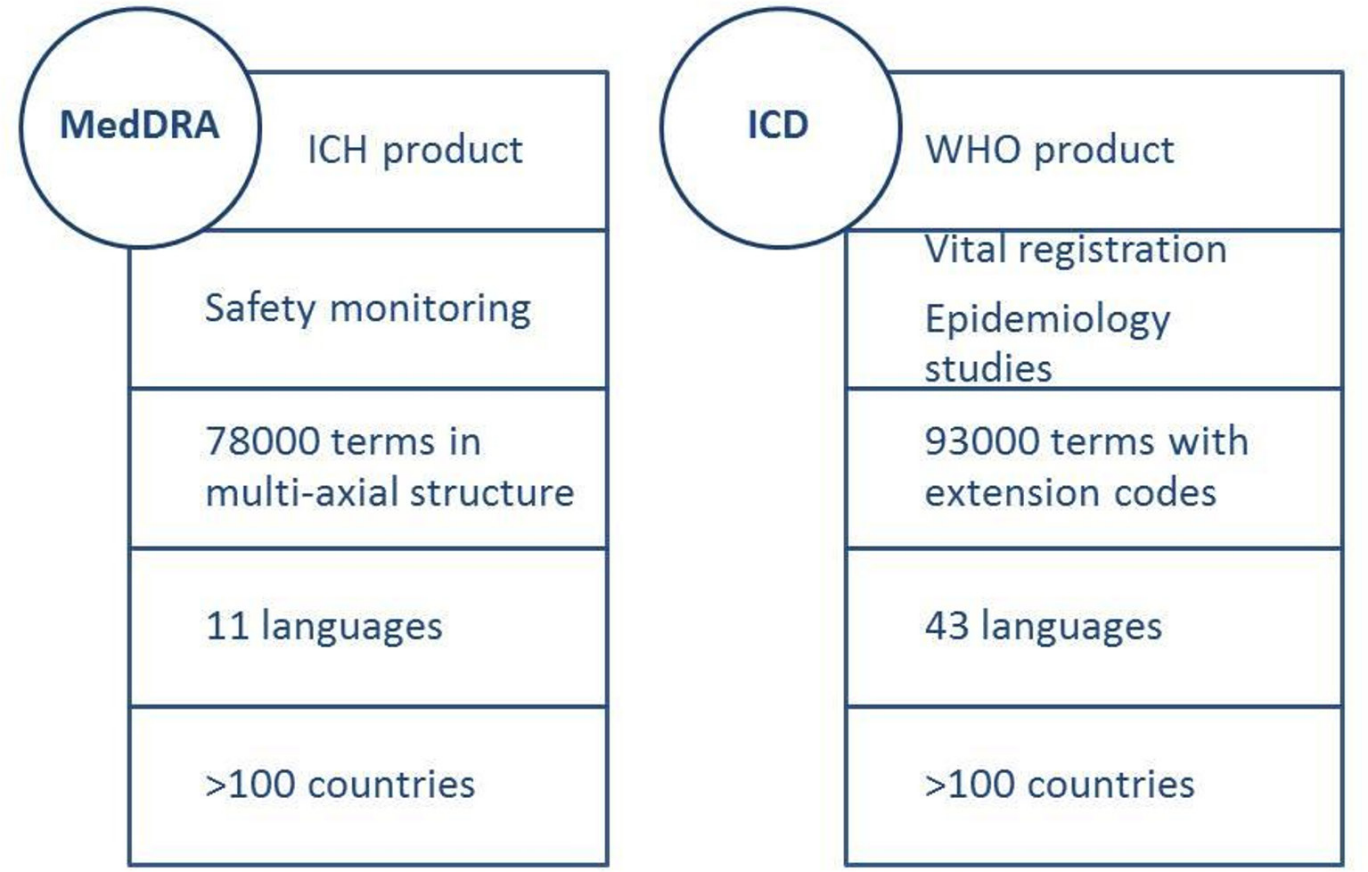
Main characteristics of MedDRA and ICD terminologies. ICD, International Classification of Diseases; ICH, International Council on Harmonisation; MedDRA, Medical Dictionary for Regulatory Activities.

Medical terminologies are controlled vocabularies that provide formal naming for entities related to biomedical science. Their use applies to numerous fields such as library or computer science and medical information architecture. Each field has developed one or several such systems. In the field of vigilance, two of the most popular ones are the Medical Dictionary for Regulatory Activities (MedDRA) in the regulatory arena and the International Classification of Diseases (ICD) in health information, planning and clinical epidemiology (figure 1).

MedDRA, developed by the International Council on Harmonisation of Technical Requirements for Registration of Pharmaceuticals for Human Use, an initiative from national regulators and pharmaceutical industries, is the reference medical dictionary for regulatory activities on health products safety monitoring from clinical development to postlicensure.^27^ MedDRA is also the terminology used by WHO’s Programme for International Drug Monitoring.^28^ It currently includes more than 78 000 terms organised through 27 system organ classes (SOC) and 4 levels of terms. Those SOCs are not mutually exclusive, and therefore a term can be represented in more than one SOC. This characteristics is referred to as multiaxiality and allows retrieving terms according to aetiology or localisation. Standardised MedDRA Queries (SMQs) allow grouping of terms across SOC and maximise the likelihood that all terms related to a specific medical condition of interest will be identified. Currently more than 100 conditions benefit from an SMQ, including several on pregnancy and neonatal topics such as congenital, familial and genetic disorders, neonatal disorders or pregnancy, and labour and delivery complications and risk factors. MedDRA is currently available in 11 languages and used in 111 countries.

ICD is WHO’s standard diagnostic tool applied to global cause-specific mortality statistics, morbidity reporting, healthcare management and clinical epidemiology. It provides a system of diagnostic codes, causes for illness and death, and reasons for health system encounters that is periodically revised. The current version, ICD-10, was issued in 1992 and underwent subsequent updates.^29^ The next version, ICD-11, has just been released in a stable version and is scheduled for adoption by the WHA in May 2019. ICD-11 includes 93 000 terms in 26 chapters, including one on pregnancy, childbirth and puerperium. It also has an extensive section for documentation of patient safety events and extension codes for substances, biologicals, anatomy, severity, temporality, injury and diagnostic criteria. Its technological disease entities have definitions to facilitate understanding. A standard definition template provides the structure for those definitions through a content model that encompasses 13 main parameters (text, body system, severity and aetiology, among others). Extension codes allow further characterising the condition according, for example, to severity, temporality, injury and diagnostic criteria. ICD-10 is currently available in 43 languages and is the most widely used dictionary globally. For ICD-11, at this stage 14 languages are in preparation. The International Classification of Diseases for Maternal Mortality^30^ is a specialised application of the ICD for statistical use with ICD-10 that identifies the conditions and codes for use as underlying cause of death during pregnancy, childbirth and puerperium. The ICD-perinatal mortality (ICD-PM) provides a similar approach for all perinatal deaths.^31^ The WHO, the US CDC and the International Clearinghouse for Birth Defects Surveillance and Research have also developed an Atlas of selected congenital anomalies,^32^ along with a facilitators’ guide. To ensure comparability of studies, a mapping exercise is under way that will allow combining studies using MedDRA or ICD.

## Verifying clinical diagnoses

Pregnancy and neonatal outcomes can reflect very diverse sets of circumstances, adding to the complexity of accurately measuring their occurrence in a reliable and comparable fashion. At a recent maternal interventions vigilance stakeholder meeting,^33^ participants examined four conditions for which recent case definitions have been proposed using a Brighton Collaboration^34^ process. These included abortion,^35^ congenital anomalies,^36^ stillbirth^37^ and preterm birth,^38^ and were selected in order to illustrate a diversity of methodological challenges in measuring pregnancy outcomes. They examined how the ability to detect, report and verify these conditions could be affected by different circumstances; what type of data are critical; possible variations in clinical presentation; the suitability of different study designs; and any other aspect that would warrant additional examination (table 1).

**Table 1:** Issues related to the measurement of four pregnancy and early childhood health outcomes

	Abortion	Congenital anomalies	Stillbirth	Preterm birth
Circumstances affecting identification	Time after conception. Access to prenatal care. Availability of pregnancy test. Cultural and legal barriers.	Very large number of conditions. Availability of diagnostic tools (systematic surface examination, prenatal, autopsy). Delayed clinical manifestation (internal anomalies, neurodevelopmental defects). Unattended home deliveries.	Masking postnatal death into stillbirth. Cultural barriers.	Contraception methods can affect identification of last menstrual period. Access and timing to prenatal care.
Data elements required for confirmation	Gestational age. Product of conception. Pregnancy test/ultrasound.	Vary with condition. Multiple case definitions needed. Availability of reference test.	Gestational age.	Gestational age.
Clinical variations	Late abortions have different aetiology.	External anomalies more likely to be promptly recognised.	Accuracy of definition depends on gestational age accuracy.	Contraception methods can affect identification of last menstrual period.
Applicability in different types of studies	Few clinical trials include first trimester pregnancies. Pregnancy registries require early identification of pregnancy. Demographic health survey sites have potential to capture cases that did not reach the healthcare system.	MedDRA and ICD used for surveillance. More detailed definitions in observational and clinical studies.	Retrospective studies and surveillance depend on availability of ICD codes. More detailed definitions in observational and clinical studies.	Prospective studies allow for accurate measurement. Retrospective studies are complicated by ascertainment of gestational age.
Aspects that require further testing and review	Gestational age assessment method and cut-off methodology. Definition of early and late abortions. Variable terminology (miscarriage, fetal loss).	Access to the infant. All available terminologies need to be evaluated in different settings.	Gestational age cut-off methodology.	Determination of gestational age in low-resource settings.

ICD, International Classification of Diseases; MedDRA, Medical Dictionary for Regulatory Activities.

This examination highlights the many issues that affect the ability to measure those important outcomes. Abortion, for example, will only be identified if a woman presents to the healthcare system and has a confirmed pregnancy. This limits significantly the ability to detect early fetal losses and probably a large number of spontaneous abortions in populations with limited access to antenatal care. In addition, cultural, legal and clinical access factors can affect the ability to document and distinguish spontaneous from elective abortions. Clinically, late abortions are affected by different causes and therefore do not represent a homogeneous entity. Studying abortions is limited by the number of pregnancies documented during the first trimester. Demographic health surveillance sites, in that respect, have the capacity of capturing a larger number of events as household visits can also identify health problems that were not captured by the healthcare system.^39^

When it comes to congenital anomalies, the main issue is that this term encompasses many diverse conditions. External anomalies are those most likely to be identified, while internal ones most often require diagnostic tools (eg, congenital heart anomalies). Depending on the availability of prenatal examinations or if autopsies are conducted, internal anomalies can be confirmed that would otherwise remain undetected (or detected later in life). Within that broad entity, many case definitions would be needed to address cutaneous, osteoarticular, cardiovascular and any other system defects, in addition to chromosomal abnormalities and other combination of defects that correspond to specific syndromes. Stillbirth can also be misrepresented, for example being misclassified as early neonatal deaths. In addition, its definition implies a distinction from abortion related to the gestational age cut-off. For those outcomes and others (preterm birth, stillbirth and small for gestational age), the ability to accurately determine gestational age is an important criterion which varies enormously depending on access to antenatal care services and the diagnostic tools available.^40–42^

## Registries for pregnancy and neonatal outcomes

Besides civil registration, general morbidity statistics and passive public health surveillance systems, two specific tracks of surveillance have been developed that are relevant for pregnancy intervention vigilance: registries for pregnancy and birth outcomes and registries for congenital birth defects. Electronic pregnancy registries present a possible solution for enhancing data availability, simplifying the number of data management systems used by different health service providers and promoting the use of harmonised terminologies. Many stakeholders are convinced that new information and communication technologies have the potential for enhancing health sector organisation towards greater efficiency and better access to care.^43^ A major challenge for maternal and child health is the fragmentation in governance and financing and multiplication of related initiatives. At least 18 maternal health initiatives have been launched in the past decade that would benefit from integrated use of information and communication technologies.^44^ The data revolution that results from greater availability of information needs to be harnessed in order to effectively enhance data use for decision making and ensure accountability of implementation.

Several networks have developed surveillance systems for congenital anomalies, including some that involve low-income and middle-income countries. The Newborn and Birth Defects Database from South-East Asia is an online system for 220 hospitals from 9 countries.^45^ The Latin American Collaborative Study of Congenital Malformations (ECLAMC)^46^ is a research programme of risk factors for congenital anomalies. The International Clearinghouse for Birth Defects Surveillance and Research^47^ is a network of institutions with members and affiliates from all continents that promotes birth defect surveillance and research.

Both pregnancy surveillance and birth defects registries have limitations. Pregnancy registry subjects are not randomly selected from a defined population. The proportion of subjects lost to follow-up, or never identified because they do not seek antenatal care, can be high. When the focus is on birth defects, data are usually of limited scale, as it is difficult and costly to actively monitor a large population given the low frequency of many birth defects. This will also affect the timeliness of data.

## Evolving needs and further integration of data: a collaborative approach

Diverse outcomes of interest, the need to monitor pregnancy and outcomes, potentially long time intervals between exposure and detectable effects, reliance on surveillance registries and observational study designs due to limited numbers of robust clinical trials, and the need to measure rare events are among the many challenges encountered in all settings. In addition, variable standards of care affect the ability to detect, verify and confirm diagnoses, as well as cultural and other differences that can substantially modify measurements depending on the outcomes of interest studied. Broadly, the current information landscape with multiplication of initiatives and tools results in loss of information where joining forces could increase the amount of data available to a larger number of stakeholders. More specifically this situation is also exemplified in the complexities associated with accurate vigilance for pregnancy outcomes as illustrated in the overview above.

The Health Data Collaborative (HDC), set up in 2015, includes over 600 global health leaders, decision makers, thought leaders and implementers from over 60 countries. They represent development partners, national governments and civil society that endorsed the Health Measurement and Accountability Post-2015 Roadmap^48^ and 5-Point Call to Action.^49^ This inclusive partnership of international agencies, governments, philanthropies, donors and academics, who share a common aim of improving health data, was set up to address a set of priority actions and targets that aim at strengthening country data and accountability systems for the post-2015 sustainable development agenda. The HDC is not a formal partnership but a way of working together based on a shared vision to strengthen country information systems to provide the data needed for better decision making and better health. The HDC framework and approach can be extended to the harmonisation of metrics and methodologies for improved vigilance for pregnancy interventions.

During clinical research diagnostic confirmation often goes beyond the standard of care, but this comes at high cost and is time-limited. Public health surveillance on the other hand relies on diagnoses based on minimal requirements. To facilitate the path to harmonisation, it is important to distinguish situations where the quantity and quality of data can be drastically different. It is proposed to develop harmonised sets of minimal data to be collected for all studies and surveillance projects of pregnant women, according to site characteristics ranging from minimal to optimal infrastructure and clinical conditions. Through the examination of case definitions applicability, the following characteristics, in combination, were identified as important:

- Healthcare infrastructure and information system capacity (minimal, intermediate and high-income setting).
- Availability of civil registration and vital statistics and/or pregnancy registries.
- Type of study (clinical trial, observational studies, public health surveillance).
- Outcome of interest (maternal/fetal/newborn/child).
- Stage of pregnancy during which interventions are being studied (first, second or third trimester (define cut-offs), first two, last two, any trimester with consideration to preconception, prepregnancy and lactation).

## Conclusions

Better approaches are needed in order to enhance the quality of vigilance data and analyses around pregnancy interventions. All pregnancy and neonatal outcomes discussed need to be measured, either to monitor progress with disease control or assess risks attributable to an intervention. To inform upcoming public health policies, they should be assessed in a comparable fashion and inform a risk–benefit equation. It is, therefore, judicious to seek harmonisation of data and tools and agree on global standards.

There is also broad agreement that the landscape of maternal, neonatal and child health would benefit from more alignment of the numerous ongoing initiatives and platforms. Leveraging and better coordinating current investment in monitoring of pregnancy outcomes is identified as an opportunity for better information. Through its normative role, WHO is well positioned to convene all stakeholders to develop and propose such standards. Subsequently, pregnancy intervention communities could join forces in order to optimise vigilance during implementation of priority interventions and measure safety and impact in a comparable fashion.
